# Swine Enteric Coronaviruses: An Updated Overview of Epidemiology, Diagnosis, Prevention, and Control

**DOI:** 10.3390/ani16030458

**Published:** 2026-02-01

**Authors:** Yassein M. Ibrahim, Can Liu, Yuandi Yu, Liu Yang, Qianlin Chen, Wenjie Ma, Gebremeskel Mamu Werid, Shaomei Li, Jie Luo, Shengbin Gao, Suhui Zhang, Lizhi Fu, Yue Wang

**Affiliations:** 1 Chongqing Academy of Animal Science, Chongqing 402460, China; yassinvet83@nyalau.edu.sd (Y.M.I.); liuc20262026@163.com (C.L.); yuyd@cqaa.cn (Y.Y.); yangl@cqaa.cn (L.Y.); qlchenjj@163.com (Q.C.); qd1992mwj@163.com (W.M.); shaomeili123@163.com (S.L.); roger0601@163.com (J.L.); zhangsuhui@cqaa.cn (S.Z.); 2 National Center of Technology Innovation for Pigs, Chongqing 402460, China; 3 Faculty of Veterinary Science, University of Nyala, Nyala 155, Sudan; 4 Rongchang Field Observation and Research Station for Animal Diseases, Ministry of Agriculture and Rural Affairs, Chongqing 402460, China; 5 Davies Livestock Research Centre, School of Animal & Veterinary Sciences, University of Adelaide, Rose Worthy Campus, Rose Worthy, Adelaide 5371, Australia; gebremeskelmamu.werid@adelaide.edu.au; 6 China Center of Animal Health and Epidemiology, Qingdao 266000, China; gaoshengbin@cahec.cn; 7 College of Veterinary Medicine, Southwest University, Chongqing 400715, China

**Keywords:** swine enteric coronaviruses, epidemiology, cross-species transmission, diagnostics, vaccines, biosecurity

## Abstract

Swine enteric coronaviruses (SECoVs), including transmissible gastroenteritis virus (TGEV), porcine epidemic diarrhea virus (PEDV), porcine deltacoronavirus (PDCoV), and swine acute diarrhea syndrome coronavirus (SADS-CoV), are major enteric pathogens causing severe diarrhea, dehydration, high neonatal mortality, and substantial global economic losses. Rapid viral evolution and recombination continually generate antigenically diverse variants that limit cross-protection and undermine vaccine efficacy, particularly for PEDV genogroup II strains that now dominate worldwide circulation. This review synthesizes current knowledge on epidemiology, diagnostic innovations, and emerging vaccine platforms, with emphasis on advances since 2022. Recent progress includes molecular surveillance tools, rapid point-of-care diagnostics, and next-generation vaccine technologies such as mRNA-based and virus-like particle platforms. However, significant knowledge gaps persist regarding viral evolution dynamics, co-infection synergies, and zoonotic spillover potential, particularly following documented human infections with PDCoV. Effective long-term control requires integrated genomic surveillance, strengthened farm-level biosecurity, rationally designed multivalent vaccines targeting conserved epitopes, and harmonized international surveillance systems to reduce outbreak risk and enhance pandemic preparedness at the human–animal interface.

## Simple Summary

Swine enteric coronaviruses are major causes of severe intestinal disease in pigs, resulting in high piglet mortality and significant global economic losses. These viruses spread rapidly between farms and evolve quickly, undermining vaccine effectiveness. This review outlines their epidemiology, emerging diagnostic tools, and available vaccines. Because some swine coronaviruses can infect other animal species and potentially humans, they also present public health concerns. Strengthening farm biosecurity, improving vaccine strategies, and enhancing genomic surveillance are essential to control these infections and safeguard both swine production and human health.

## 1. Introduction

Swine enteric coronaviruses (SECoVs), including porcine epidemic diarrhea virus (PEDV), transmissible gastroenteritis virus (TGEV), porcine deltacoronavirus (PDCoV), and swine acute diarrhea syndrome coronavirus (SADS-CoV), are highly contagious pathogens that threaten pig health and cause substantial economic losses in the global swine industry [1]. These viruses primarily infect intestinal epithelial cells, leading to villous atrophy and enterocyte loss. Clinically, infection manifests as severe enteritis characterized by anorexia, vomiting, watery diarrhea, dehydration, lethargy, metabolic acidosis, hyperkalemia, and high mortality in piglets [2,3]. Although clinically similar, SECoVs are antigenically distinct and confer no cross-protection [4,5]. Coronaviruses (CoVs) belong to the subfamily *Orthocoronavirinae* within the family *Coronaviridae* (order *Nidovirales*) [6], and are taxonomically divided into four genera: *Alphacoronavirus* (α-CoV), *Betacoronavirus* (β-CoV), *Gammacoronavirus* (γ-CoV), and *Deltacoronavirus* (δ-CoV). (https://ictv.global/report/chapter/coronaviridae/coronaviridae [ICTV, 2019], accessed on 22 November 2025). *Gamma*- and *Deltacoronaviruses* primarily infect birds, whereas alpha- and betacoronaviruses infect a wide range of mammals, including humans, livestock, rodents, bats, and other wild animals, causing diseases of the respiratory, digestive, hepatic, and nervous systems [7].

**Figure 1:**
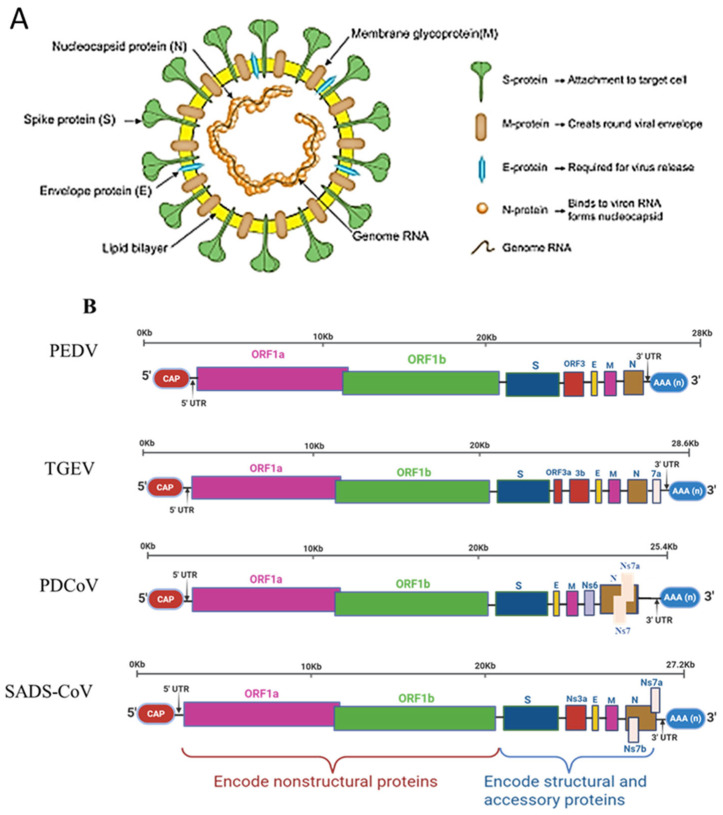
Ge. me organization and structure of SECoVs. (**A**) Schematic of porcine coronaviruses’ (PEDV, TGEV, PDCoV, and SADS-CoV) genome structure showing virions containing S, E, and M proteins in the lipid bilayer, enclosing the N protein–RNA complex (gray line) [19]. (**B**) Genome map showing structural proteins (S, E, M, N) and accessory genes (Ns3a, Ns6, Ns7, Ns7a, Ns7b), with a 5′ cap and 3′ poly(A) tail.

Coronavirus virions are spherical to pleomorphic, measuring 80–120 nm in diameter, and are characterized by trimeric spike (S) glycoproteins that form a distinctive crown-like structure under electron microscopy [6,7] (Figure 1A). The coronavirus genome consists of positive-sense, single-stranded RNA of 26–32 kb that encodes structural proteins (spike, envelope [E], membrane [M], and nucleocapsid [N]), non-structural proteins (nsp1–16), and multiple accessory proteins [6]. The accessory gene composition varies among SECoVs: TGEV encodes NS3a, NS3b, and NS7; PEDV encodes ORF3; PDCoV encodes NS6, NS7, and NS7a; and SADS-CoV encodes NS3, NS7a, and NS7b (Figure 1B). The S protein mediates viral entry, with the S1 subunit recognizing host cell receptors and determining tissue tropism, while S2 enables membrane fusion [6,8]. The S protein also harbors key epitopes essential for neutralization, vaccine design, diagnostics, and T-cell responses. The M, E, and N proteins support virion assembly, structural integrity, and host immune modulation, while non-structural proteins drive genome replication and evade antiviral defenses [8,9,10]. Accessory proteins, though dispensable for replication in vitro, contribute substantially to virulence and immune evasion in vivo [11].

SECoVs exhibit high mutation and recombination rates, particularly within the S gene, resulting in quasispecies diversity, which enables rapid adaptation, immune escape, and cross-species transmission [12,13]. Their genomic plasticity accelerates viral evolution and raises concerns regarding potential zoonotic spillover events [14]. Historical outbreaks demonstrate the devastating impact of SECoVs on global pig production: in 2010, China reported over one million piglet deaths [15]; in 2013, the United States experienced more than eight million fatalities with estimated economic losses of USD 900 million to 1.8 billion [16,17]; and in 2017, the first outbreak of SADS-CoV in China caused more than 20,000 piglet deaths [18].

Although several reviews on SECoVs have been published in recent years, most focus primarily on epidemiology, genomic variation, or phylogenetic classification, with limited integration of technological and translational advances. This review synthesizes recent progress (post-2020) in diagnostics, vaccine development, and next-generation sequencing-based genomic surveillance, bridging molecular evolution, epidemiology, and control strategies. It further offers a comparative synthesis of viral recombination and interspecies transmission, with particular emphasis on the emergence of recombinant and cross-species SECoV strains.

## 2. Epidemiology Occurrence and Transmission

### 2.1. Pathogenesis and Clinical Manifestations

TGEV has long circulated in pigs, whereas PEDV, PDCoV, and SADS-CoV are emerging or re-emerging coronaviruses that continue to threaten global pig health and production. SECoVs primarily replicate in the jejunum and ileum, where they induce interferon responses and apoptosis [20,21]. Infection leads to severe intestinal epithelial damage characterized by villous atrophy, mucosal thinning, and impaired nutrient absorption, resulting in diarrhea, dehydration, and weight loss [22,23].

Clinical signs are similar across SECoVs, appearing 1–3 days post-exposure, lasting up to 10 days, and often followed by extended viral shedding that enables recovered pigs to act as reservoirs [24]. Disease severity is strongly age-dependent [24]. Neonatal piglets are most susceptible: TGEV and PEDV can cause nearly 100% mortality, SADS-CoV is highly fatal (>90%) in piglets <5 days old, while PDCoV typically induces milder disease with 30–40% mortality in neonates [25]. Older pigs, especially sows, generally develop mild symptoms with low mortality. Clinical outcomes depend on host factors such as immature intestinal barriers and limited neonatal immunity, as well as viral factors including recombination and antigenic variation [25]. Co-infections, environmental stress, and high viral loads further aggravate disease severity [25,26]. Epidemiological patterns and mortality data are summarized in Table 1.

**Table 1:** Global epidemiology parameters of major swine enteric coronaviruses since 2000.

Genus	Virus	First Detection/Major Outbreaks	Key Prevalence Trends	Morbidity	Mortality of Piglets	Geographic Distribution	Notable Recent Findings	Reference
α-CoV	TGEV	1946, USA; widespread mid-20th century	Now rare in Europe/USA; sporadic in Asia (i.e., China, Korea)	Less than 3%	Up to 100% in neonates	Declining globally, persists sporadically in Asia	Decline linked to PRCV cross-protection; a low-pathogenic mutant providing partial immunity	[27,28,29]
α-CoV	PEDV	1971, UK; 2010 China resurgence (>1 M piglets’ death); 2013 U.S. epidemic (>8 M piglets lost)	Persistent high prevalence in Asia (6–90%); lower but recurrent in Europe/North America	80–100%	50–100%	Asia, Europe, Americas; sporadic in Africa	G2 strains dominate globally; vaccine mismatch persists; recombinant strains reported	[15,29,30,31,32,33]
δ-CoV	PDCoV	2012, Hong Kong; 2014, USA outbreaks	Prevalence generally lower (1–30%), localized peaks >60% (e.g., Thailand 2015)	20–30%	30–40%	Asia, Americas; not reported in Europe/Africa	Serology suggests broader exposure; China meta-analysis ~14% positivity; detection in children in Haiti 2021.	[34,35,36]
α-CoV	SADS-CoV	2017, Guangdong, China (~24,500 piglet deaths)	Low PCR detection (<5%); serology shows widespread exposure (60–80% in China)	About 10%	90–100% in piglets <5 days	China; related viruses in Vietnam bats/pigs; 2024	Strong bat origin evidence; re-emergence in 2019 & 2021; zoonotic potential	[37,38,39]

### 2.2. Historical Context and Global Distribution

**Figure 2:**
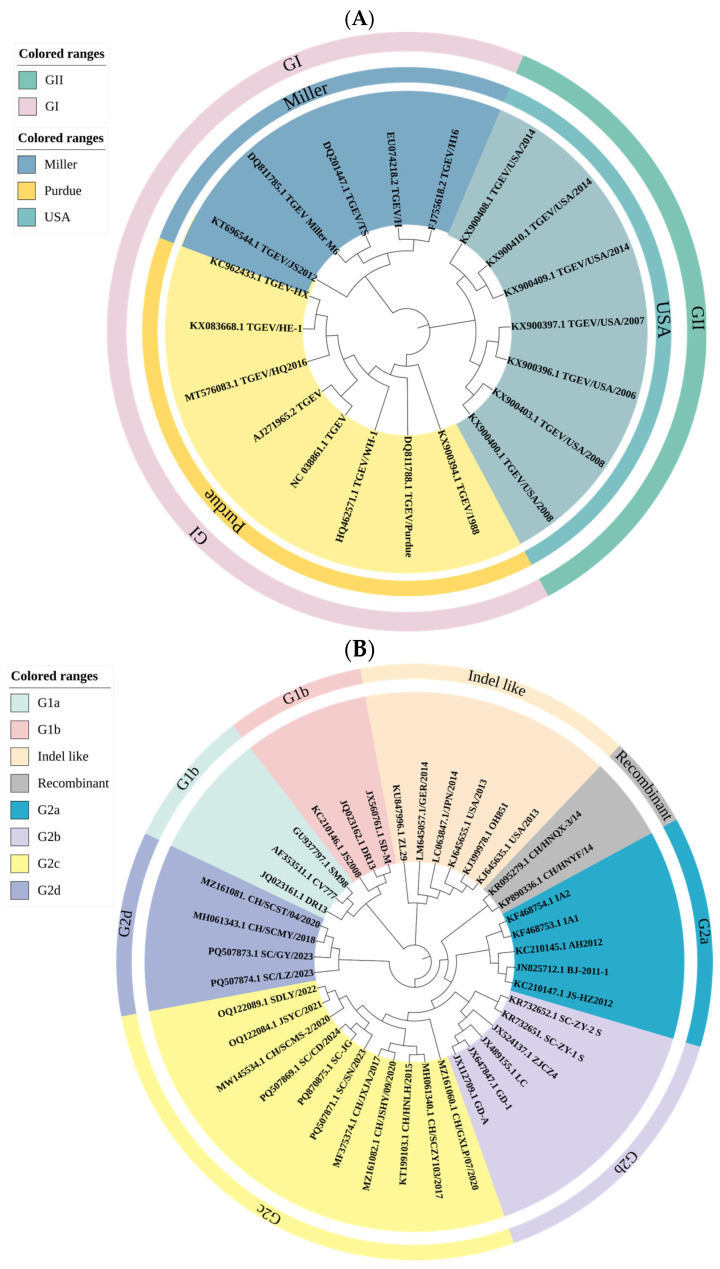

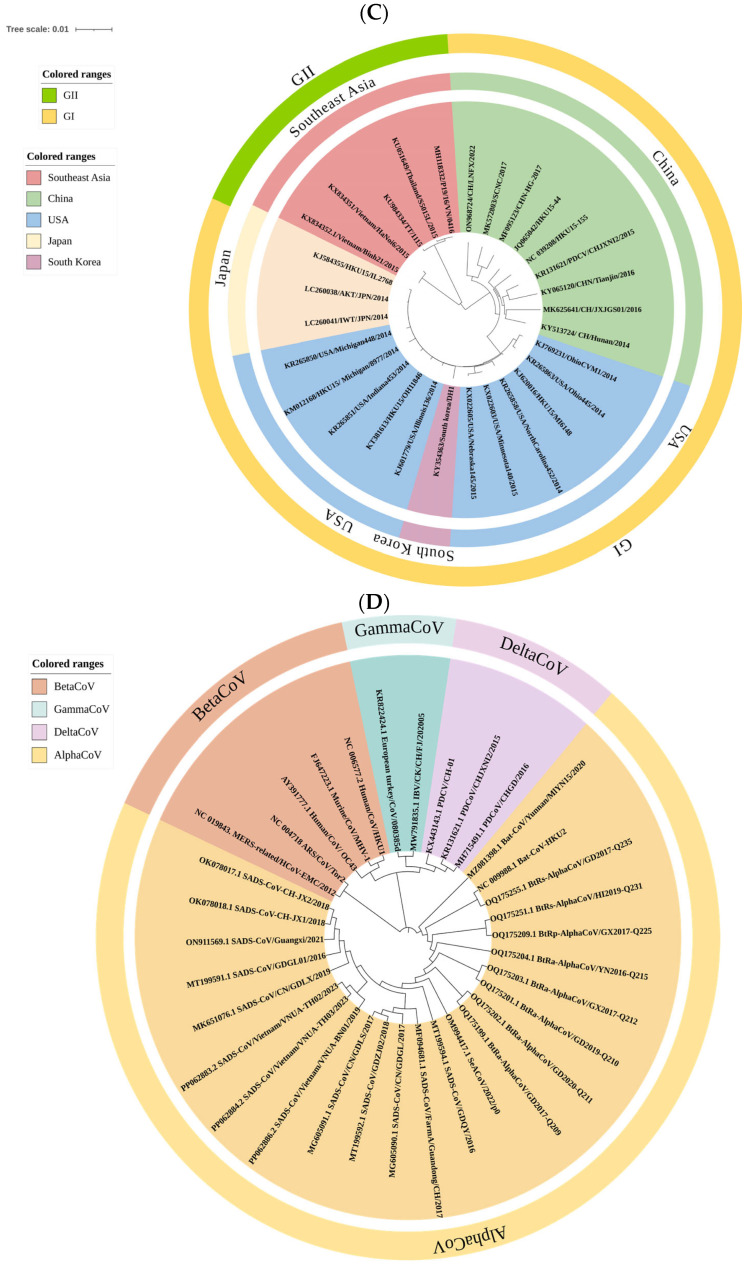
Phylogenetic analyses of swine enteric coronaviruses. (**A**) Whole-genome phylogeny of TGEV constructed in MEGA 7.0, showing two subtypes (GI and GII) and three major lineages (Purdue, Miller, and U.S. variants). (**B**) Phylogenetic analysis of PEDV spike (S) gene using the neighbor-joining method in MEGA 7.0. Clades are color-coded: G2a (green), recombinant (gray), G2c (yellow), G2b (blue), G1a (pink), and G1b (red). (**C**) PDCoV S protein phylogeny showing strains clustered into GI and GII lineages from China, the United States, Japan, and Korea. (**D**) SADS-CoV S gene phylogeny generated in MEGA 7.0, with SADS-CoV strains highlighted in red. All sequences were retrieved from the NCBI database.

TGEV was first identified in the United States in 1946 [40] and subsequently spread globally [41]. Until the 1980s, it remained a major cause of enteric disease despite vaccination. Currently, TGEV prevalence has markedly declined, largely due to the widespread circulation of porcine respiratory coronavirus (PRCV) [42], a naturally occurring TGEV variant carrying a 621–681 nt deletion in the spike gene that attenuates virulence and alters tropism from enteric to respiratory tissues. PRCV generally causes mild or subclinical respiratory infections but induces cross-neutralizing antibodies that confer partial protection against TGEV, reducing selective pressure for virulent enteric strains [43]. Despite lacking major antigenic sites, PRCV retains conserved epitopes that cause strong serological cross-reactivity with TGEV [28,44]. Consequently, PRCV-induced antibodies often yield false-positive results in standard TGEV ELISAs and neutralization assays, complicating serodiagnosis and obscuring true epidemiological patterns unless differential or molecular assays are used [43]. TGEV is classified into two genotypes: GI (including Purdue and Miller strains) and GII (comprising U.S. variants) [45,46], Figure 2A. Despite reduced prevalence, new virulent strains have emerged, including recombinant Purdue–Miller variants in China [47,48]. In Europe, a recombinant swine enteric coronavirus (SeCoV) derived from TGEV and PEDV was reported, possessing an S gene >90% identical to PEDV and the remaining genome up to 97% identical to virulent TGEV strains [49,50]. Additionally, virulent isolates such as JS2012, HQ2016, and SC2021 continue to appear sporadically in Asia and the Americas [48,51,52].

PEDV was first reported on British farms in 1971, and the CV777 strain identified in Belgium in 1978 became the classical PEDV reference [30]. During the 1970s–1980s, PEDV caused major outbreaks across Europe, leading to serious economic losses. From the late 1980s to 2010, the virus circulated mainly in Europe and Asia, causing sporadic or localized outbreaks [31]. A highly virulent variant emerged in China in 2010, spreading rapidly and causing catastrophic losses even in vaccinated herds, highlighting antigenic mismatch and vaccine failure [31]. In 2013, closely related strains introduced into the United States triggered an epidemic, reducing the national swine population by approximately 3%, with losses of USD 900 million to 1.8 billion [53]. By late 2016, genetically diverse PEDV lineages had become established across North America, Asia, and parts of Europe. Today, PEDV remains a persistent and re-emerging threat to swine health and production in most pig-producing regions worldwide [32,54].

Phylogenetic analysis defines two major genogroups: GI (classical) and GII (variant), comprising six subgroups (GIa, GIb, GIIa, GIIb, GIIc, GIId) [55,56,57,58], Figure 2B. GI includes early virulent European strains; GII contains variants that emerged around 2010 and now dominate globally, particularly in China and North America [56,59,60]. The recombination-driven GII lineage continues to evolve rapidly and remains poorly controlled by GI-based vaccines [33,60].

PDCoV was first detected in Hong Kong in 2012 [61]. Its pathogenic significance became evident in 2014 when it caused major outbreaks in Ohio that spread to >20 U.S. states [62,63]. Since then, PDCoV has been reported across North America, Asia, and South America [64,65], resulting in notable economic losses. PDCoV causes 30–40% mortality in neonatal piglets, increasing sharply during co-infections [29]. Phylogenetically, PDCoV comprises two genogroups: GI (China, America, Japan, Korea) and GII (Southeast Asia), including SEA-1 (Thailand), SEA-2 (Vietnam), and the recombinant SEA-2r strain [65], Figure 2C. Chinese isolates display the highest genetic diversity, positioning China as a principal evolutionary reservoir [65,66].

SADS-CoV was first identified in Guangdong, China, in 2017, causing outbreaks with approximately 90% case fatality rates in piglets <5 days old [18,37,67]. Whole-genome sequencing shows exceptional nucleotide identity (approximately 99.9%) among isolates from 2017 to 2023 [39,68], indicating minimal genetic diversification. Phylogenetic analyses show that all strains cluster with HKU2-like coronaviruses in bats, sharing 95–98% nucleotide identity [18,69], strongly supporting bat origin and likely a single spillover event followed by sustained pig-to-pig transmission [70]. The spike gene exhibits unexpected affinity to betacoronaviruses despite SADS-CoV being classified as an alphacoronavirus, a pattern also observed in some rodent α-CoVs [71], suggesting historical recombination or ancestral divergence between α- and β-CoVs [37,72,73], Figure 2D. Although currently confined to China, the detection of related viruses in Vietnamese bats and pig feces [68,74] indicates the potential for cross-border spread.

### 2.3. Transmission of SECoVs

**Figure 3:**
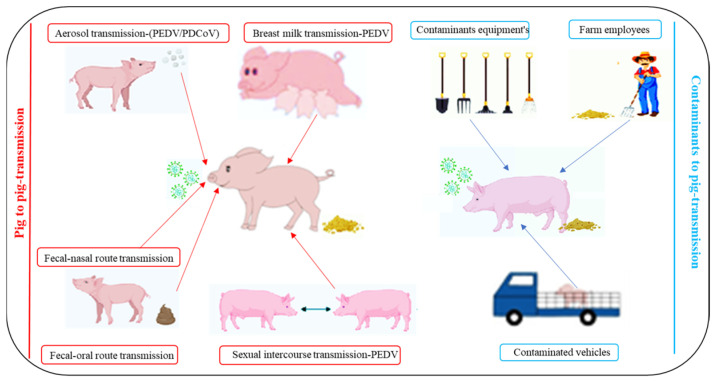
Transmission routes of SECoV infection.

Understanding SECoV transmission is essential for effective control. These viruses spread primarily via the fecal–oral route and indirectly through contaminated equipment, feed, personnel, and trailers [24,75,76,77]. PEDV and PDCoV can also transmit through aerosols [32,34], with PEDV RNA detected up to 10 km from infected farms [78,79]. PEDV RNA has been detected in the semen of infected boars with prolonged shedding in the sperm-rich fraction [80], indicating potential sexual transmission. Additionally, PEDV can be transmitted lactogenically; virus-bearing T cells migrate to the mammary gland and are secreted in colostrum and milk [81], providing a clinically significant route that sustains herd-level infections. Milk-borne viral shedding post-infection exposes entire litters at their most susceptible stage, bypassing biosecurity measures and potentially overwhelming maternal antibody protection [81]. Figure 3 summarizes the major transmission pathways of SECoV. The environmental resilience of SECoVs facilitates prolonged survival and sustained indirect transmission between farms.

### 2.4. Global Prevalence and Distribution

SECoV prevalence is associated with sampling season, region, year, pig stage, and clinical signs [82,83]. Geographic variation reflects differences in climate, production systems, biosecurity capacity, and surveillance intensity. Outbreaks are more frequent in winter and spring [1], when prolonged viral survival and increased indoor housing enhance transmission. Large commercial systems experience frequent detection via active surveillance, whereas smallholder regions report sporadic outbreaks due to limited diagnostic infrastructure [84]. International trade in pigs and feed, along with shared transport networks, facilitates cross-border transmission and the emergence of novel variants [85,86]. The rapid global spread of PDCoV after its 2014 emergence in the U.S. illustrates this transboundary risk [24]. Table 2 summarizes SECoV prevalence by country.

TGEV prevalence varies globally, but accurate estimation is hindered by extensive serological cross-reactivity with PRCV, which has circulated widely across Europe, North America, and Asia since the mid-1980s [43,48,51,52]. Studies lacking differential diagnostic methods often overestimate true TGEV exposure [28,87]. For example, the Canadian study reporting 7.2% seropositivity [88] did not differentiate PRCV, and concurrent circulation suggests some positives were PRCV-derived. Similarly, a 2020 Chinese survey reporting 54% seropositivity [89] likely reflected combined TGEV/PRCV antibody responses given PRCV’s endemic status [90]. In contrast, molecular surveillance using RT-PCR and S gene sequencing consistently reports very low TGEV detection (<1%), indicating that TGEV is now rare while PRCV remains widespread [91,92]. In the U.S., analysis of 29,397 samples (2008–2016) found 2.3% positivity overall, peaking at 6.8% in 2010 and declining to <0.1% by 2014 [42]. In Japan, TGEV caused eight outbreaks between 2001 and 2007, with 14.4% seroprevalence in 2010 but no detections after 2008 [93,94]. A 2023 South Korean study similarly found 4.3% seropositivity without active infection [95]. Overall, TGEV circulation appears sporadic. Seroprevalence data should be interpreted cautiously unless PRCV differentiation is confirmed, and future surveillance should combine molecular assays with validated differential serological tests.

PEDV spread widely through Europe and Asia in the 1990s [31,96], and re-emerged in China in 2010 [31], followed by the U.S. (2013) [97], Mexico (2013) [98], and Canada (2014) [99]. Subsequent outbreaks across South Korea, Thailand, Vietnam, and India [31,100], along with renewed activity in Europe [101,102,103,104,105,106,107], caused major losses. Piglet mortality reached approximately 30% in Hungary [108] and >70% in Germany [109]. In Croatia, mortality reached 20–30% in suckling piglets, with 82.8% seropositivity at the farm level [110]. In China, extensive surveillance (2011–2023) documented persistent PEDV circulation, with prevalence ranging from 6.0% to 92.7% depending on diagnostic method and sample type [32,33,55,92,111,112,113]. PEDV remains the dominant SECoV (>45% detection), requiring continuous surveillance and genotype-specific vaccines.

PDCoV prevalence from 2011 to 2023 varied substantially across Asia and the Americas. Moderate detection was reported in South Korea (19.0%) [114], Japan (15.1%) [64], Vietnam (10.2%) [115], and Mexico (9.6%) [116]. In the U.S., prevalence reached 25–30.4% in 2014 [117], with 244 outbreaks reported from 2015 to 2023 across 16 states as incidence increased from 0.44% to 4.28% [118]. A severe outbreak in Thailand in 2015 caused 27.6% mortality in sows and 64.3% in piglets, with 86.7% positivity [119]. In China, detection rates ranged from 1.2% to 69.5% depending on province and study design, while serological studies recorded 11–65% antibody positivity [91,92,120,121,122,123]. More recent nationwide surveys (2020–2023) consistently reported lower prevalence (1.2–14.1%) [92,123,124,125,126], suggesting declining circulation.

Molecular detection of SADS-CoV from 2012 to 2024 varied widely across studies. Large fecal surveys reported low molecular detection (0.23–2.2%) [91,92], whereas targeted sampling showed higher rates (15–43.5%) [127,128]. Serology revealed higher exposure (59.9–81.7%, 2020–2023) [89,129]. The detection of related viruses in Vietnamese bats and pigs (7.2%) [68,74] supports cross-border transmission.

SADS-CoV has been reported mainly in China and Vietnam, likely due to bat reservoir distribution, intensive pig production, and wildlife–livestock interfaces [18,67,69], with phylogenetic evidence indicating dissemination from China to Vietnam [68,130].

Under-surveillance, misdiagnosis, and biosecurity differences may explain the lack of reports elsewhere [131]. Therefore, expanded molecular and serological surveillance in regions with high bat diversity and intensive pig production is essential to clarify the global distribution and emergence risk of SADS-CoV.

**Table 2:** Geographic distribution and prevalence of swine enteric coronaviruses by country and period.

Virus	Country	Period	No. of Samples	Sample Type	Positive Samples	Type of Test	Prevalence Rate	References
TGEV	Canada	1990	305	Serum	22	ELISA	7.2%	[88]
	Belgium	1990	160	Serum	12	ELISA	7.5%	[132]
	USA	2008–2016	29,397	Fecal	667	qPCR	2.3%	[27]
	Hungary	2015–2016	908	Serum	140	IFA	15.4%	[28]
	Italy	2016–2017	444	Serum	3	ELISA	0.67%	[133]
		2022	438	Serum	24	ELISA	5.5%	[134]
	Poland	2021–2024	828	Serum	18	ELISA	2.2%	[45]
	Spain	2017–2018	215	Fecal	6	PCR	2.8%	[135]
	Germany	2000–2005	1194	Serum	19	ELISA	1.59%	[136]
	Czech Republic	1999–2005	134	Serum	1	ELISA	0.75%	[137]
	Croatia	2005–2009	556	Serum	2	ELISA	0.4%	[138]
	Argentina	2014–2017	87	Serum	3	ELISA	3.4%	[139]
	Cuba	2008	90	Intestine	9	PCR	10%	[140]
	Japan	2007–2010	2703	Serum	389	ELISA	14.4%	[93]
	South Korea	2010	1295	Serum	64	ELISA	4.9%	[141]
		2023	350	Serum	15	ELISA	4.3%	[95]
	China	2012–2014	314	Fecal	6	PCR	1.9%	[142]
		2015	44	Intestine	8	nPCR	18.2%	[143]
		2015–2016	114	Fecal	4	qPCR	3.5%	[144]
		2012–2016	390	Fecal	6	PCR	1.5%	[145]
		2015–2016	27	Intestine	5	PCR	18.5%	[146]
		2016–2018	181	Fecal	1	mPCR	0.55%	[147]
		2015–2018	543	Fecal	46	PCR	8.5%	[148]
		2017–2018	672	Fecal	35	qPCR	5.2%	[149]
		2012–2018	2987	Fecal	21	PCR	0.7%	[91]
		2019–2021	176	Fecal + Intestine	12	PCR	6.8%	[150]
		2020	300	Serum	162	SiELISA	54%	[89]
		2021–2023	1791	Fecal	15	qPCR	0.84%	[92]
		2022–2023	5483	Fecal + Intestine	11	qPCR	0.2%	[124]
PEDV	Italy	2007–2014	51	Fecal	38	PCR	74%	[101]
		2016–2017	444	Serum	17	ELISA	3.8%	[133]
		2022	438	Serum	65	ELISA	14.8%	[134]
		2007–2014	51	Fecal	47	ELISA	92%	[101]
	Vietnam	2011–2016	108	intestine	87	PCR	80.6%	[115]
	Spain	2017–2018	215	Fecal	5	PCR	2.3%	[135]
		2017–2019	106	Fecal	41	PCR	38.7%	[151]
	China	2011–2012	288	Fecal + Intestine	267	PCR	92.7%	[152]
		2011–2012	577	Intestine + Milk	417	PCR	72.3%	[153]
		2011–2021	149,869	Fecal	70,384	PCR	46.9%	[33]
		2012–2014	314	Fecal	76	PCR	24.2%	[142]
		2012–2015	356	Fecal + Intestine	231	nPCR	64.9%	[120]
		2014–2015	129	Intestine	119	PCR	92.3%	[154]
		2014–2018	645	Fecal+ Intestine	231	PCR	35.8%	[112]
		2015–2016	252	Fecal	165	PCR	65.5%	[155]
		2012–2016	390	Fecal	88	PCR	22.6%	[145]
		2015–2016	114	Fecal	55	qPCR	48.2%	[144]
		2015–2016	27	Intestine	25	PCR	92.6%	[146]
		2015–2017	398	Fecal	78	mPCR	19.6%	[121]
		2015–2018	543	Intestine	363	PCR	66.9%	[148]
		2015–2019	575	Fecal + Intestine	297	PCR	51.7%	[156]
		2016–2017	116	Fecal + Intestine	61	PCR	52.6%	[111]
		2016–2017	170	Fecal + Intestine	133	PCR	78.2%	[127]
		2016–2018	719	Fecal	267	qPCR	36.7%	[122]
		2016–2018	181	Fecal	56	mPCR	30.9%	[147]
		2012–2018	2987	Intestine	1712	PCR	57.3%	[91]
		2017–2018	672	Fecal	128	qPCR	19.1%	[149]
		2017–2019	634	Fecal + Intestine	204	qPCR	32.2%	[157]
		2019–2020	413	Fecal + Intestine	257	PCR	62.2%	[158]
		2019–2021	176	Fecal + Intestine	66	PCR	37.5%	[150]
		2020	300	Serum	18	SiELISA	6%	[89]
		2017–2021	882	Fecal	564	qPCR	63.9%	[57]
		2017–2022	673	Fecal + Intestine	363	PCR	53.9%	[159]
		2020–2022	548	Fecal	258	PCR	47.1%	[160]
		2021–2022	112	Intestine	58	qPCR	51.8%	[125]
		2021–2023	1791	Fecal	890	qPCR	49.7%	[92]
		2022–2023	481	Fecal	341	PCR	71%	[126]
		2022–2023	5483	Fecal + Intestine	485	qPCR	8.8%	[124]
	India	2022–2023	339	Serum	18	ELISA	5.3%	[100]
	Japan	2013–2014	1269	Fecal	248	PCR	19.5%	[161]
		2013–2014	204	Fecal	148	PCR	72.5%	[162]
	South Korea	2013–2022	1131	Fecal + Intestine	140	PCR	12.4%	[163]
	Mexico	2016–2018	68	Fecal	53	qPCR	77.9%	[164]
		2019–2021	1054	Serum	650	ELISA	61.7%	[165]
		2021	74	Swab	50	PCR	67.6%	[166]
	Vietnam	2019	600	Serum	533	ELISA	88.8%	[167]
	Thailand, Vietnam, Philippines and Lao PDR	2015	97	Fecal	82	PCR	84.5%	[168]
	Philippines	2014–2015	34	Fecal	10	PCR	29.4%	[169]
	Croatia	2016	397	Serum	62	ELISA	15.6%	[110]
PDCoV	South Korea	2014–2016	683	Fecal	130	nPCR	19.0%	[114]
	USA	2014	293	Fecal	89	qPCR	30.4%	[117]
		2014	435	Fecal	109	qPCR	25%	[118]
	Japan	2013–2014	477	Fecal	72	qPCR	15.1%	[64]
	Vietnam	2011–2016	108	Intestine	11	PCR	10.2%	[115]
	Mexico	2014–2017	885	Swab	85	mPCR	9.6%	[116]
	Thailand	2015	30	Different	26	PCR	86.7%	[119]
	Thailand, Vietnam, Philippines and Lao PDR	2015	97	Fecal	12	PCR	12.4%	[168]
	China	2014–2015	192	Fecal	73	LAMP	38%	[170]
		2015–2016	252	Fecal	55	PCR	21.8%	[155]
		2015–2016	871	Serum	96	ELISA	11%	[171]
		2012–2015	356	Fecal + Intestine	120	nPCR	33.7%	[120]
		2012–2016	390	Fecal	5	PCR	1.3%	[145]
		2015–2017	430	Fecal	101	PCR	23.5%	[172]
		2015–2017	398	Fecal	144	mPCR	36.2%	[121]
		2015–2018	543	Fecal	26	PCR	4.8%	[148]
		2016–2017	170	Fecal + Intestine	15	PCR	8.8%	[127]
		2012–2018	2987	Fecal	813	PCR	27.2%	[91]
		2016–2018	719	Fecal	94	qPCR	13.1%	[122]
		2016–2018	181	Fecal	32	mPCR	17.7%	[147]
		2017–2018	672	Fecal	26	qPCR	3.87%	[149]
		2017–2019	177	Fecal + Intestine	123	PCR	69.5%	[173]
		2017–2019	634	Fecal + Intestine	84	qPCR	13.3%	[157]
		2020	300	Serum	196	SiELISA	65.3%	[89]
		2020–2023	4897	Fecal	362	mqPCR	7.4%	[123]
		2021–2022	112	Intestine	3	qPCR	2.7%	[125]
		2021–2023	1791	Fecal	307	qPCR	14.1%	[92]
		2021–2023	481	Fecal	10	PCR	2.1%	[126]
		2022–2023	5483	Fecal + Intestine	67	qPCR	1.2%	[124]
SADS-CoV	China	2012–2018	2987	Fecal	7	PCR	0.23%	[91]
		2016–2017	170	Fecal + Intestine	74	PCR	43.5%	[127]
		2016–2018	181	Fecal	21	mPCR	11.6%	[147]
		2020	300	Serum	245	SiELISA	81.7%	[89]
		2021–2023	1791	Fecal	40	qPCR	2.2%	[92]
		2022–2023	12,978	Serum	7783	ELISA	59.9%	[129]
		2024	72	Fecal + Intestine	11	qPCR	15.3%	[128]
	Vietnam	2018–2023	69	Fecal	5	PCR	7.2%	[130]

### 2.5. Co-Infections Dynamics Underlying Conditions

Co-infection with enteric pathogens complicates SECoV control [148] by obscuring diagnosis and increasing morbidity and mortality in neonatal piglets [174,175]. Enterocytes in the jejunum and ileum are frequently co-infected by PEDV, PDCoV, TGEV, and SADS-CoV, creating ideal conditions for recombination and the emergence of novel variants [42,174,176]. SECoV co-infections are widely reported across Asia and the Americas, with common combinations including PEDV/PDCoV, PEDV/TGEV, and PEDV/SADS-CoV, as shown in Table 3.

PDCoV/PEDV co-infection reached 29.9% in Southeast Asia (2015) and 1.1–9.9% in South Korea, Mexico, and the USA [114,116,117,168]. In China (2012–2023), rates ranged from 0.1% to 19.66%, dominated by PEDV/SADS-CoV (17.7%) [127], and PDCoV/PEDV (19.66%) [120]. Moderate prevalence was observed for PEDV/TGEV (0.22–11.1%) [91,92,144,146,174] and PDCoV/TGEV (5.9%) [172]. Large-scale surveillance (2012–2018) reported PEDV/PDCoV co-infection in 12.72% of 2987 samples, while other combinations were <0.3% [91].

Co-infections exacerbate disease severity by impairing cytokine responses, damaging gut integrity, and enhancing susceptibility to secondary pathogens. For example, PEDV facilitates SADS-CoV infection [127]. Such interactions have likely contributed to the emergence of new SECoVs, including the recent chimeric TGEV-PEDV virus in Europe [61,62,101,177,178]. SECoVs co-circulate with porcine rotaviruses A and C, increasing disease severity and shedding [125,179] and highlighting the need for multiplex diagnostic assays.

**Table 3:** Co-infection with swine enteric coronaviruses across periods and regions.

Period	Country	Pathogens	Samples No.	Coinfection Rate	Host	Reference
2021–2023	China	PEDV/PDCoV	1791	41(2.29)	Swine	[92]
		PEDV/TGEV		5 (0.28)		
		PEDV/SADS-CoV		4 (0.22)		
2018–2021	China	PEDV/PDCoV	4468	1.50%	Swine	[174]
		PEDV/TGEV		1.17%		
2016–2018	China	PDCoV/PEDV	719	34 (4.73%)	Swine	[122]
2016–2018	China	PEDV/PDCoV	181	17 (9.4%)	Swine	[147]
		PEDV/SADS-CoV		13 (7.2%)		
2015–2018	China	PDCoV/PEDV	430	61(14.2%)		[172]
		PDCoV/TGEV		6 (5.9%)		
2015–2018	China	PDCoV/PEDV		(4.96%)	Swine	[148]
2013–2018	China	PDCoV/PEDV	1547	17 (1.1%)	Swine	[34]
2012–2018	China	PDCoV/PEDV	2987	380 (12.72%)	Swine	[91]
		PEDV/TGEV		9 (0.3%)		
		PEDV/SADS-CoV		3 (0.1%)		
		PDCoV/TGEV		3 (0.1%)		
2015–2016	China	PDCoV/PEDV	252	2 (3.6%)	Swine	[155]
2015–2016	China	PEDV/TGEV	114	3 (2.6%)	Swine	[144]
2015–2016	China	PEDV/TGEV	27	3 (11.1%)	Swine	[146]
2016–2017	China	PDCoV/PEDV		3 (1.59%)	Tibetan pigs	[34]
2016–2017	China	PEDV/SADS-CoV	170	30 (17.7%)	Swine	[127]
2017–2019	China	PEDV/PDCoV	634	47 (7.4%)	Swine	[157]
2016–2017	China	PDCoV/PEDV	172	16 (9.3%)	Swine	[180]
2017	China	SAD-CoV/PEDV	170	30 (17.65%)	Swine	[127]
2014–2017	Mexico	PDCoV/PEDV PDCoV/TGEV PDCoV/PEDV/ TGEV	885	46 (5.2%) 9 (1.1%) 16 (1.8%)	Swine	[116]
2014–2016	South Korea	PDCoV/PEDV	683	43 (6.3%)	Swine	[114]
2012–2016	China	PDCoV/PEDV	420	25 (5.95%)	Swine	[34]
2012–2016	China	PDCoV/PEDV	390	5 (1.28)	Swine	[145]
2011–2016	Vietnam	PDCoV/PEDV	108	11 (10.19%)	Swine	[115]
2014	USA	PDCoV/PEDV	435	19 (4.37%)	Swine	[181]
2014	USA	PDCoV/PEDV	293	29 (9.9%)	Swine	[117]
2015	Thailand, Vietnam, Philippines and Lao PDR	PDCoV/PEDV	97	29 (29.9%)	Swine	[168]
2012–2015	China	PDCoV/PEDV	356	70 (19.66%)	Swine	[120]

### 2.6. Cross-Species Transmission and Zoonotic Risks

Coronaviruses exhibit high mutation rates (10^−3^ to 10^−5^ substitutions per site per replication cycle) and recombination frequencies despite the proofreading activity of nsp14 (ExoN) [182,183], generating quasispecies that enhance adaptability [12,13,184]. This enables rapid host adaptation, immune evasion, and cross-species transmission [185,186,187]. This genomic plasticity accelerates viral diversification and zoonotic potential. The animal origins of SARS-CoV, MERS-CoV, and SARS-CoV-2 [188], combined with widespread coronavirus circulation in birds and mammals and their rapid evolutionary dynamics [182,189,190], underscore the public health relevance of SECoVs, which share ancestral lineages with these zoonotic pathogens.

TGEV can infect wild and domestic carnivores (foxes, dogs, mink, cats) subclinically, enabling reservoir maintenance, although only canine-derived strains have been confirmed to infect pigs [49]. A novel canine coronavirus (CCoV-HuPn-2018) identified in Malaysian pneumonia patients, mainly children with animal exposure, showed high genetic similarity to canine coronaviruses and virulent TGEV [191]. Phylogenetic analysis clustered the virus with canine, feline, and porcine alphacoronaviruses, including TGEV and SeCoV [191], confirming close evolutionary relationships.

Evidence for PEDV cross-species transmission is scarce. The virus has been identified in wild pigs only in Korea and the United States, likely reflecting spillover from domestic herds [192,193]. However, PEDV can replicate in human, monkey, pig, and bat-derived cell cultures, supporting the hypothesis that it previously crossed species barriers, most plausibly from bats to pigs [194,195].

SADS-CoV exhibits broad host tropism, with efficient replication in cell lines derived from swine, poultry, cattle, felines, non-human primates, and humans [196,197,198,199,200,201,202,203,204,205], suggesting intrinsic viral determinants that facilitate cross-species infection. Accordingly, despite negative serological findings among occupationally exposed farm workers [18], subsequent experimental studies demonstrated significant zoonotic potential [204], as the virus replicates efficiently in primary human respiratory and intestinal cells via ACE2/DPP4/CD13-independent entry mechanisms [204].

PDCoV demonstrates the most robust cross-species transmission capacity among SECoVs. Experimental infections show productive replication and transmission in mice, calves, and poultry [39,201,206,207], while natural infections have been documented in wild birds, Asian leopard cats, and badgers [61]. In vitro studies reveal broad cellular tropism across multiple mammalian and avian lineages, including human intestinal epithelial cells [2,208]. Most significantly, three PDCoV strains were isolated from children presenting with acute febrile illness in rural Haiti [209], providing direct evidence of natural human infection in settings characterized by close livestock contact. These findings highlight the evolutionary adaptability of SECoVs and implicate swine as potential intermediate hosts for emerging zoonotic coronaviruses. Proactive One Health surveillance, integrating molecular monitoring in swine systems with seroepidemiological screening of exposed populations, is essential for early spillover detection and pandemic preparedness [210].

## 3. Diagnostic Approaches

SECoV diagnosis is challenging due to overlapping clinical signs, requiring reliable laboratory confirmation [25,211]. Traditional methods such as virus isolation, immunofluorescence (IF), immunohistochemistry (IHC), electron microscopy, and polymerase chain reaction (PCR) are informative but slow and unsuitable for field use [29]. Molecular diagnostics now serve as the primary tools for SECoV detection, offering higher sensitivity, faster turnaround, and improved capability to identify co-infections [212]. A detailed summary of available diagnostic platforms, including their advantages and limitations, is presented in Table 4.

PCR-based assays, including multiplex RT-PCR, RT-qPCR, nested RT-PCR, digital droplet PCR (ddPCR), and nanoparticle-assisted PCR (NanoPCR), offer high sensitivity and detect co-infections, though they require specialized equipment and extensive validation [41,121,144,147,179,213,214,215,216,217]. These platforms enable simultaneous detection of multiple pathogens, facilitating rapid differential diagnosis in complex clinical scenarios.

Isothermal methods such as recombinase-aided amplification (RAA) and recombinase polymerase amplification (RPA) (LAMP) provide rapid, portable detection and perform best when paired with microfluidics, lateral-flow strips, or CRISPR systems [218,219,220]. These approaches are particularly valuable for field deployment and resource-limited settings. CRISPR-Cas12a/Cas13a assays enable rapid, sensitive, and multiplex detection but remain costly and are not yet field-optimized [3,220,221,222,223,224]. These emerging platforms show considerable promise for point-of-care applications once scalability and cost challenges are addressed.

Serology, particularly ELISA, remains essential for herd surveillance and vaccine monitoring, although it is less effective for early piglet detection [211]. Advances include phage-ELISAs targeting M protein for TGEV differentiation, recombinant protein ELISAs for PEDV, M protein assays for PDCoV IgG, and multiplex immunoassays using recombinant S1 proteins that allow simultaneous detection of PEDV, TGEV, and PDCoV [171,225,226,227,228]. Rapid immunochromatographic strips (ICTs) offer significant advantages in point-of-care testing due to their brief testing duration, user-friendliness, and cost-effectiveness [229,230,231]. Histopathology combined with immunohistochemistry is valuable for confirming lesions and visualizing viral antigens in tissues, providing definitive diagnostic evidence in outbreak investigations.

Although isothermal amplification, sequencing, and nanotechnology offer promising advances, many diagnostics lack cross-strain validation. CRISPR-based methods need optimization, affordable portable tools are scarce, and next-generation sequencing is not yet widely applied in routine surveillance. Future priorities should focus on developing sensitive, cost-effective, field-ready diagnostics that enable earlier detection of emerging SECoV strains and facilitate real-time epidemiological monitoring.

**Table 4:** Advantages and limitations of diagnostic assays used for swine enteric coronaviruses.

Method	Examples	Advantages	Limitations	Applications	References
Traditional	Virus isolation, IF, IHC, electron microscopy	Visual confirmation; gold standard for lesions	Slow, labor-intensive, not field-suitable	Confirmation of pathology, research	[29,232]
Conventional PCR	RT-PCR, nested RT-PCR	Specific, reliable	Time-consuming; contamination risk	Routine lab detection	[29]
qPCR/multiplex RT-PCR	RT-qPCR, multiplex RT-PCR	High sensitivity; detects co-infections; quantification	Requires expensive equipment	Surveillance, clinical diagnosis	[41,179,216,217,233,234,235]
Advanced PCR	ddPCR, DPO-PCR, NanoPCR	Ultra-sensitive; quantifies low-load samples	High cost; technical complexity	Precision diagnostics; mixed infections	[144,147,229]
Isothermal amplification	RAA, RPA, LAMP	Rapid, low-equipment; field-friendly	Limited validation; risk of false positives	Point-of-care screening	[218,219,220].
CRISPR-based detection	Cas12a, Cas13a	Rapid, highly sensitive; multiplexing	Cost; requires optimization	Future field diagnostics; rapid outbreak detection	[3,220,221,222,223,224].
Serology	ELISA, phage-ELISA, recombinant ELISA	Herd monitoring; vaccine evaluation	Not suitable for early piglet infection	Serosurveillance, vaccine studies	[171,211,225,226,227,228]
Rapid antigen/antibody tests	Lateral-flow strips	Simple, immediate	Lower sensitivity	Farm-level rapid screening	[229,230,231]
Sequencing	NGS, amplicon sequencing	Detects variants; surveils evolution	Expensive; limited routine use	Genomic surveillance, emerging strain detection	[236]

## 4. Prevention and Control Measures

### 4.1. Current Vaccine Strategies

Current vaccination strategies for SECoVs primarily target pregnant gilts and sows to confer passive lactogenic immunity to neonatal piglets via colostrum and milk [237]. However, rapid mutation and frequent recombination within SECoV genomes have progressively reduced vaccine effectiveness [12,13,238], a challenge exacerbated by the emergence of PDCoV and SADS-CoV. Although the PEDV spike glycoprotein contains major neutralizing epitopes (COE, SS2, SS6, and 2C10) [239,240,241], extensive amino acid variation in contemporary G1b and G2 strains compromises antibody recognition [33]. Consequently, classical CV777-derived vaccines provide strong protection against homologous strains but limited efficacy against currently circulating G2 variants [242,243]. A comparative summary of key vaccine studies for SECoVs prevention, including methods, immune responses, efficacy, and limitations, is provided in Table 5.

Inactivated and Live-Attenuated Vaccines remain the primary platforms for TGEV and PEDV [32,243], while no licensed vaccines are available for PDCoV or SADS-CoV [123,244]. Live-attenuated vaccines induce robust immunity but pose safety concerns due to potential reversion via mutation or recombination [49]. Recent advances include ORF3-truncated GIIb PEDV strains, which elicit strong IgG and IgA responses in sows and provide partial cross-protection in piglets [245], as well as reverse-genetics-derived attenuated vaccines with deletions of interferon-antagonistic genes that improve genetic stability [246].

Inactivated vaccines are highly safe but weakly immunogenic, requiring booster doses and potent adjuvants. Parenteral administration induces limited mucosal and lactogenic immunity, restricting neonatal protection [49]. Adjuvant optimization has shown promise; for example, a ginseng-derived saponin mucosal adjuvant enhanced intestinal IgA responses and milk IgA levels when combined with inactivated PEDV vaccines [247]. Nevertheless, cross-protection against heterologous strains remains inconsistent.

In 2025, autogenous inactivated vaccines prepared from tissues infected with the PEDV G2c variant were rapidly deployed on farms, resulting in a 95% reduction in mortality and viral shedding. Genomic sequencing confirmed antigenic matching with circulating strains and showed no evidence of reversion risk [237,248].

Next-Generation Vaccine Platforms, including mRNA, virus-like particle (VLP), subunit, and vectored vaccines, aim to improve safety, mucosal immunity, and breadth of protection [96,243,244]. lipid nanoparticles-formulated mRNA vaccines enable rapid, flexible development and strong immunogenicity [249]. PEDV GIIb spike or S-M mRNA constructs elicited broad neutralizing antibodies (GI, GIIa, GIIb) in animal models and reduced disease severity in piglets [250], though viral shedding was not fully prevented.

VLP Vaccines: VLPs expressing PEDV E, M, and S induce strong mucosal and systemic immunity and reduce disease severity [251]. HEK293T- and baculovirus-derived VLPs elicit higher IgA and neutralizing antibodies than inactivated vaccines [252,253,254,255], supporting their potential as safe and effective SECoV platforms.

Subunit vaccines: Efforts focus on recombinant spike proteins and neutralizing domains. Bivalent S-trimer vaccines overcome limited GIIa/GIIb cross-protection, inducing strong lactogenic immunity and protection against both lineages [256]. Stabilized S-trimers and M103-adjuvanted S formulations elicit broad neutralizing responses and reduce disease and viral shedding in piglets [257,258]. Strain-matched GIIb vaccines further boost maternal titers, with pre-farrowing levels (~1:377–1:774) associated with >80% piglet protection [259].

Live bacterial/viral vectors: Oral recombinant Lactobacillus expressing PEDV S antigens induces strong mucosal and systemic immunity in sows, elevating milk SIgA and providing effective lactogenic protection [260,261]. In contrast, Adenoviral (Ad5) vectors encoding S or S1 elicit robust humoral responses after intramuscular delivery but have limited efficacy via the oral route [262].

For PDCoV, most vaccine studies are still in the preclinical stage. Highlighted among advances are mRNA plus nanoparticle-based, scalable, broad-spectrum protective coverage type future candidates [263]. Live attenuated PDCoV vaccine development has focused on genetic modification and serial passaging [52]. In 2020, an NS6-deficient mutant (rPDCoV-ΔNS6-GFP) was identified as a promising candidate [264], causing only mild clinical signs compared with the wild-type virus, indicating NS6 as a key virulence factor. This mutant was generated using an infectious cDNA clone of strain USA/IL/2014/026, enabling precise genetic engineering.

Inactivated vaccines provide partial lactogenic protection but fail to fully prevent disease [265]. Emerging platforms, including ferritin-based RBD nanoparticles, baculovirus-expressed subunit vaccines, and VLPs, induce robust immune responses and protection comparable to inactivated vaccines in experimental models [266,267,268].

Given the pronounced genetic and antigenic diversity of SECoVs, Polyepitopic vaccines incorporating conserved epitopes represent a promising strategy for achieving broader cross-protective immunity, supported by advances in in silico epitope design, though validation in swine remains essential [269].

**Table 5:** Comparative analysis of vaccine platforms developed for the prevention of swine enteric coronaviruses (2020–2025).

Virus Target	Vaccine Type	Expression System/Platform	Antigen	Adjuvant	Animals Model	Vaccine Efficacy	Limitations	Route of Administration	Reference
TGEV	Subunit	*E. coli*	S protein epitopes (in silico predicted)	–	In silico	Predicted non-toxic, highly immunogenic, stable, and cost-effective epitopes	Experimental validation required (in vitro/in vivo)	–	[270]
TGEV	Subunit	*E. coli* K12	S protein epitopes (in silico)	–	In silico	Predicted immunogenic epitopes	Requires experimental verification	–	[271]
PEDV	Live attenuated	Serial in vitro passages	ORF3-deleted GIIb strain	–	Piglets	Immunogenicity and protective efficacy in pigs	Effects of mutations on pathogenicity require further investigation	IM	[245]
PEDV	Live attenuated (chimeric construction)	Reverse genetic S2 domain replaced with DR-13 (G1) sequence	YN144 (G2)	-	piglets	Broad-spectrum: Neutralizes both G1 and G2 strains.	Preclinical	PO	[272]
PEDV	Live attenuated backbone engineering	Reverse genetic modification of polymerase/ genome structure	PC22A (G2b)	-	piglets	Prevents recombination between vaccine and wild- type strains.	Preclinical	PO	[273]
PEDV	Live attenuated CRISPR/Cas9	Replacement of TRS-S with TRS- M	AJ1102/G2	-	piglets	Enhanced viral gene expression and antigen production.	Preclinical	PO	[274]
PEDV	Inactivated	Formaldehyde inactivation	PEDV ShXXY2-2023	ISA 201	Piglets	Induced neutralizing antibodies and protective immunity; virus-neutralizing activity in vitro	Additional animal studies required	IM	[259]
PEDV	Subunit (plant-based)	Plant expression system	CO-26K-equivalent epitope (COE)	Water-in-oil	Piglets	High PEDV-specific IgG, IgA, neutralizing antibodies, and IFN-γ responses	Optimization of dose and adjuvant; shedding studies required	IM	[275]
PEDV	Subunit	Insect cells and silkworm	S protein trimer	Montanide IMS 1313	Mice	Immunogenicity in mice and virus-neutralizing activity of mouse sera in vitro	No pig challenge model	IM	[257]
PEDV	Subunit	HEK 293F cells	S protein trimer, S1 subunit, COE	M103 or M401	Mice, piglets	Immunogenicity in mice and pigs, virus-neutralizing activity of mouse sera in vitro and protective efficacy in pigs		IM	[258]
PEDV	Subunit	HEK-293F cells	S protein trimer (GIIa and GIIb)	M103	piglets	Immunogenicity and protective efficacy in pigs and virus-neutralizing activity of swine sera in vitro	Preclinical	IM	[256]
PEDV	Subunit	Trimer-Tag technology subcloned into the pPink-HC expression vector and expressed in yeast	S1 subunit trimer, COE trimer, RBD trimer	IAS 201	Mice, sows	Immunogenicity in mice and pigs, virus-neutralizing activity of mouse and swine sera in vitro and protective efficacy in pigs	Preclinical	IM	[276]
PEDV	Subunit	CSFV vector expressing S1	S1 protein	–	Mice	High neutralizing antibody titers, IgG1/IgG2a, and IL-4 induction	No pig challenge model	IM	[277]
PEDV	Subunit	CHO cells	Spike ectodomain	Montanide, IAS 201 VG	piglets	Immunogenicity and protective efficacy in pigs and virus-neutralizing activity of swine sera in vitro	Preclinical	IM	[278]
PEDV	Bacterial vector	*Lactobacillus casei*	S1 glycoprotein	–	Mice	Strong humoral and cellular immune responses after oral administration	Protective efficacy in pigs not evaluated	PO	[261]
PEDV	Bacterial vector	*Lactobacillus acidophilus*	S1 and S2 proteins	–	Mice, sows	Induced systemic and mucosal immune responses in mice and pregnant sows	Challenge studies lacking	PO	[279]
PEDV	Bacterial vector	*Lactobacillus paracasei* (genome-integrated)	S1 protein	–	Mice, piglets	Immunogenic and protective efficacy in pigs	Limited field validation	PO	[250]
PEDV	Bacterial vector	*Lactobacillus casei*	PEDV S + Brucella OMP16	Freund’s complete adjuvant	Mice	Elevated IgG, neutralizing antibodies, IL-4, IL-10, IFN-γ	No pig challenge	PO	[280]
PEDV	VLP	Insect cells	S, M, E proteins	Freund’s adjuvant + CCL25/CCL28	Pigs	Protective immunity and neutralizing antibodies in pigs	Preclinical	IM	[251]
PEDV	VLP	HEK293T cells	S, M, E proteins	Alum	Mice	Immunogenic with virus-neutralizing antibodies	Protective efficacy not evaluated	IM	[252]
PEDV/TGEV	Chimeric VLP (ADDomer)	High Five cells	TGEV S1 + PEDV S1/S2 epitopes	ISA 201VG	Piglets	Immunogenic with neutralizing antibodies	Preclinical	IM	[255]
PEDV	Viral vector (Adenovirus 5)	HEK293 cells	S and S1 (GIIa/GIIb)	–	Mice	Immunogenic and neutralizing antibodies	No pig challenge	IM/PO	[262]
PEDV	Viral vector (Adenovirus 5)	HEK293 cells	COE-GIIb	–	Mice	Immunogenic and neutralizing antibodies	No pig challenge	IM/IN	[281]
PEDV	Viral vector (Adenovirus 5)	HEK293 cells	S-GIIb	–	Piglets	Immunogenic and protective efficacy in pigs	Preclinical	IM/IN	[282]
PEDV	Viral vector (pseudorabies virus)	ST cells	S1 epitopes	–	Piglets	Protective immunity and neutralizing antibodies	Vector safety concerns	IM	[283]
PEDV	Viral vector (BVDV)	–	S fragment (aa 499–602)	–	Mice	PEDV- and BVDV-specific IgG induction	No pig challenge	PO	[284]
PEDV	Viral vector (CSFV)	–	S1 NCOE	–	Rabbits, piglets	Elevated PEDV antibodies and IFN-γ	Preclinical	IM/IN	[285]
PEDV	Viral vector (PoRV)	Reverse genetics	ORF3 replaced with VP7	–	Piglets	Dual PEDV and PoRV neutralizing antibodies	Preclinical	IM/PO	[286]
PEDV	Viral vector (PRRSV)	–	S-G2	–	Piglets	Dual immunity against PEDV and PRRSV	Safety and recombination concerns	IM	[287]
PEDV	DNA vaccine	pPink-HC vector	S1-, COE-, and RBD-trimers	–	Mice, sows, piglets	Strong systemic and mucosal IgG/IgA responses	Not commercialized	IM	[276]
PEDV	mRNA-LNP	In vitro transcription	S or SM	–	Mice, piglets	Protective immunity and neutralizing antibodies	Cold chain and cost constraints	SC/IM	[288]
PDCoV	Inactivated	β-propiolactone	HNZK-02 strain	Al (OH)3 or ODN2395	Mice	Strong humoral and cellular immunity	Low mucosal immunity vs. LAV	SC	[224]
PDCoV	Inactivated vs. subunit	–	Whole virus vs. spike protein	–	Piglets, sows	Long-term antibodies and reduced shedding	Model-dependent	IM	[289]
PDCoV	Live attenuated	NS6 deletion	USA/IL/2014/026	–	Piglets	Reduced virulence, stable attenuation	Reversion risk	PO	[264]
PDCoV	Live attenuated	Serial passage CZ2020	CZ2020	–	Piglets	Reduced virulence, improved gut microbiota	Variant-specific, preclinical	PO	[52]
PDCoV	Live attenuated	Serial passage DHeB1	DHeB1	–	Piglets	Safe, reduced lesions and viral load	Field validation needed	IM	[290]
PDCoV	VLP	HEK293F cells	S, M, E proteins	M103	Mice, sows, piglets	Protective immunity and neutralizing antibodies	Adjuvant-dependent	IM/PO	[268]
PDCoV	VLP	Insect cells	S, M, E proteins	Freund’s	Mice	Immunogenic	No pig challenge	IM	[254]
PDCoV	Subunit	Insect cells	RBD	Al (OH)3, CpG2395, aqueous	Mice	Immunogenic	No pig challenge	IM	[291]
PDCoV	Subunit	*E. coli*	RBD dimer	Ferritin + ISA 201VG	Mice	Protective immunity and neutralizing antibodies	Preclinical	IM	[266]
PDCoV	Subunit	Insect cells	S1 protein	Gel 01	Mice, sows	Protective immunity	Preclinical	IM	[267]
PDCoV	Subunit	CHO cells	S, N, M proteins	AddaVax	Mice, piglets	Immunogenic	Preclinical	IM	[292]
PDCoV	Viral vector (PRV, CRISPR/Cas9)	–	S protein	–	Mice	Local and systemic immunity	Vector-related safety issues	IM	[293]
PDCoV	Nanoparticle	*E. coli*/HEK293F	S1-CTD	–	Mice, sows, piglets	Robust and prolonged protective immunity	Cost and scalability issues	IM	[294]
PDCoV	mRNA-LNP	In vitro transcription	S2P stabilized S	–	Piglets	Strong humoral and cellular immunity	Cold chain, limited trials	PO	[263]
PDCoV	mRNA-LNP	In vitro transcription	S and S ectodomain	–	Mice, piglets	Protective immunity and neutralizing antibodies	Cold chain, limited trials	IM	[295]

### 4.2. Therapeutic and Antiviral Drugs

SECoV control remains limited by significant antigenic variation and gaps in ecological understanding [238]. The absence of licensed vaccines for several SECoVs and the modest efficacy of existing antivirals [3] underscore the urgent need for safe, effective therapeutics. Although hundreds of compounds have been screened, most exhibit single-mechanism activity with uncertain clinical relevance [3].

The main protease (Mpro) is highly conserved across PEDV, TGEV, PDCoV, and SADS-CoV [3], making it an attractive antiviral target. A summary of bioactive compounds with anti-SECoV activity and their proposed mechanisms of action is provided in Table 6. Several candidates that show promise include: Rhodamine derivative LJ001, which inhibits TGEV and PDCoV replication [296], while melatonin and related indole compounds (indole, tryptamine, L-tryptophan) impair viral entry and replication [297]; Rifampicin, which shows activity against PEDV and SADS-CoV [3]; Griffithsin, which blocks PEDV attachment and PDCoV entry through S-protein binding [298,299]; Surfactin, which inactivates TGEV and disrupts PEDV membranes [300,301]; and 25-hydroxycholesterol, which suppresses all major SECoVs and SARS-CoV-2 [157,302,303]. Ergosterol peroxide blocks PEDV and PDCoV entry, reduces apoptosis, and modulates cytokines through NF-κB and p38/MAPK pathways [304,305]. Lithium chloride (LiCl) suppresses PEDV and PDCoV replication and reduces apoptosis [306,307]. Octyl gallate (OG) is a 3CLpro inhibitor that protects piglets from PEDV in vivo [308]. In addition, RNA interference targeting PDCoV N or PEDV/SADS-CoV M genes effectively inhibits viral replication [309]. Drug repurposing of FDA-approved compounds provides a rapid, cost-effective strategy for identifying clinically relevant therapies. Host-targeted interventions are especially promising: for example, bile acids differentially modulate SECoV replication [310], enhancing PEDV [311], inhibit PDCoV [312], and promoting SADS-CoV via lipid raft-mediated endocytosis [313]. Such findings highlight critical virus-host differences that can guide rational antiviral design.

Natural products remain appealing antiviral candidates due to their low toxicity and reduced risk of resistance. Although systematic screening, structure–activity analyses, and compound optimization show promise, most findings are confined to in vitro studies. Validation in advanced models, such as organoids, 3D cultures, and piglet challenge systems, is still needed. Combination therapies targeting both viral and host pathways may further enhance therapeutic efficacy. Future research should integrate functional genomics, high-throughput screening, and computational drug design with rigorous preclinical evaluation to accelerate the development of safe, effective antivirals, an essential component of sustainable swine production.

**Table 6:** Summary of bioactive compounds with anti-SECoV activity and their proposed mechanisms of action.

Compound	Target Virus	Testing Model	Antiviral Mechanism	Main Phases of Action	Reference
Rhodamine derivative LJ001	TGEV	In vitro	Interferes with the replication stage of viral life cycle	Replication	[296]
	PDCoV	In vitro	Interferes with the replication stage of viral life cycle	Replication	[296]
Melatonin	TGEV	In vitro	Blocks viral entry	Replication	[297]
	PEDV	In vitro	Blocks viral entry		[297]
	PDCoV	In vitro	Blocks viral entry		[297]
Indole	TGEV	In vitro	Blocks viral entry	Replication	[297]
	PEDV	In vitro	Blocks viral entry		[297]
	PDCoV	In vitro	Blocks viral entry		[297]
Rifampicin	PEDV	In vitro	Inhibits viral replication	Replication	[314]
	SADS-CoV	In vitro	Inhibits viral replication		[314]
Griffithsin	PEDV	In vitro	Inhibits viral attachment and disrupts cell-to-cell transmission of virions	Attachment	[298]
	PDCoV	In vitro	Inhibition of porcine reproductive and respiratory syndrome virus replication by rifampicin in vitro. attachment and internalization by binding to S protein	Attachment	[299]
Surfactin	TGEV	In vitro and In vivo	Acts on viral lipids to inhibit fusion between viral and cellular membrane	Attachment, entry	[300]
	PEDV	In vitro and In vivo	Acts on viral lipids to inhibit fusion between viral and cellular membrane		[300]
25-hydroxycholesterol	TGEV	In vitro	Blocks viral entry	Attachment, entry	[302]
	PEDV	In vitro	Blocks viral entry	Attachment, entry	[302]
	PDCoV	In vitro	Blocks viral entry	Attachment, entry	[315]
Ergosterol peroxide	PEDV	In vitro	Inactivates virions directly and inhibits viral internalization, replication and release Alleviates PEDV-induced apoptosis	Entry, replication, release	[304]
	PDCoV	In vitro and In vivo	Inhibits viral attachment, entry, and the early and middle phases of post-entry stage Alleviates apoptosis and elevated p38 phosphorylation	Attachment, entry, Replication	[305]
Lithium chloride	TGEV	In vitro	Inhibited virus attachment	Entry, replication	[220]
	PEDV	In vitro	Inhibited virus attachment	Entry, replication	[306]
	PDCoV	In vitro	Inhibited virus attachment	Entry, replication	[307]
Octyl gallate	PEDV	In vitro and In vivo	Interacts with PEDV 3CLpro and inhibits 3CLpro activity	Replication	[308]

### 4.3. Biosecurity Measures

Biosecurity refers to coordinated management and hygiene preventing pathogens from entering or spreading within livestock production [84,316]. No single measure is effective in isolation; protection relies on integrating multiple practices. Effective SECoV control requires understanding their diverse transmission routes. SECoVs spread primarily via the fecal-oral route, but PEDV and PDCoV also transmit through aerosols [32,34], and PEDV can spread lactogenically [81]. PEDV RNA detection in boar semen, with prolonged shedding in the sperm-rich fraction [80], suggests possible sexual transmission. Indirect transmission via contaminated feed, equipment, personnel, and transport trailers further amplifies risk [24,75,76,77]. Lactogenic and sexual routes are particularly problematic, as high viral loads in milk and semen can bypass standard biosecurity and undermine maternal antibody protection.

Critical practices:

Effective control of SECoVs relies on strict biosecurity measures that regulate the movement of animals, feed, vehicles, equipment, and personnel to limit pathogen introduction and spread. Facility sanitation should be strengthened through clearly defined Lines of Separation and Perimeter Buffer Areas, farm-specific clothing and boots, and handwashing or shower-in/shower-out protocols. Proper manure management, including fermentation or disinfection, and restrictions on staff and equipment movement between barns are critical to reducing indirect transmission. Feed and water supplies must be microbiologically safe, while transport vehicles and loading facilities, major sources of SECoV dissemination [24], require thorough cleaning and disinfection. Additional internal biosecurity measures, such as excluding wildlife and pets, routine disinfection and waste disposal, increased farm spacing, and reduced pig density, further limit viral circulation. Although controlled feedback exposure of pregnant sows to feces from infected piglets may enhance lactogenic immunity via the gut-mammary sIgA axis [317], this practice remains poorly standardized, produces inconsistent outcomes, and carries significant biosafety risks [32,191].

Implementation gaps remain substantial. Large commercial farms generally apply multi-layered biosecurity that reduces but does not eliminate-risk, whereas many smallholder systems lack fundamental measures such as effective waste management, air control, and movement restrictions, enabling viral persistence and transmission. Shared transportation further amplifies exposure risk [318]. Strengthening SECoV control will require updated farm-level data, risk-based biosecurity strategies tailored to production systems, and targeted producer training to address both structural and behavioral barriers.

## 5. Challenges and Future Perspectives

Despite extensive research on swine enteric coronaviruses, long-term control remains challenging due to genetic diversity, antigenic drift, and limited cross-protection among circulating strains. Conventional vaccines and surveillance systems often lag behind viral evolution, underscoring the need for real-time genomic monitoring and innovative immunization strategies. Integrating high-throughput sequencing, bioinformatics-driven epitope prediction, and advanced vaccine platforms (e.g., mRNA, viral vectors, and virus-like particles) represents a paradigm shift in swine coronavirus control.

Major challenges include spike-driven immune escape, antigenic divergence between PEDV genogroups (G1/G2), inadequate induction of mucosal IgA and lactogenic immunity, variable field performance of diagnostic tools, and persistent biosecurity gaps, particularly in smallholder production systems.

Research priorities include:

- Developing predictive models of viral evolution for real-time surveillance and early warning;
- Advancing broad-spectrum mucosal vaccines and delivery platforms to induce durable cross-protection and lactogenic immunity;
- Standardizing immunization evaluation frameworks with harmonized efficacy criteria;
- Implementing rapid, field-deployable diagnostics for early detection and strain differentiation;
- Elucidating host–pathogen interactions, including viral entry, immune evasion, and age-related susceptibility;
- Strengthening One Health-integrated surveillance to mitigate interspecies transmission risks.

Coordinated global surveillance at the human–animal interface is essential to track viral evolution, guide vaccine updates, and enhance preparedness for emerging coronavirus threats.

## 6. Conclusions

Integrated genomic surveillance, rapid field-deployable diagnostics, and next-generation vaccine platforms are essential for improving the control of swine enteric coronaviruses. Priorities include the development of broadly protective multivalent and mucosal vaccines, enhanced biosecurity and maternal immunization strategies, and real-time monitoring of infection dynamics and immune responses. Given the zoonotic potential of PDCoV and the broad cellular tropism of SADS-CoV, deeper mechanistic studies of pathogenicity and accelerated development of broad-spectrum antivirals and universal coronavirus vaccines are urgently needed. Proactive One Health surveillance frameworks that integrate animal and human health systems will be critical for early spillover detection and strengthened pandemic preparedness.

## Data Availability

This study is based on the analysis of publicly available documents and published literature. No new datasets were generated or analysed. Data sharing is not applicable to this article.
